# Correcting misperceptions of exponential coronavirus growth increases support for social distancing

**DOI:** 10.1073/pnas.2006048117

**Published:** 2020-06-24

**Authors:** Joris Lammers, Jan Crusius, Anne Gast

**Affiliations:** ^a^Psychology Department, University of Bremen, 28359 Bremen, Germany;; ^b^Social Cognition Center Cologne, University of Cologne, 50923 Cologne, Germany

**Keywords:** coronavirus, exponential growth bias, statistical literacy, comparison

## Abstract

The most effective way to stem the spread of a pandemic such as coronavirus disease 2019 (COVID-19) is social distancing, but the introduction of such measures is hampered by the fact that a sizeable part of the population fails to see their need. Three studies conducted during the mass spreading of the virus in the United States toward the end of March 2020 show that this results partially from people’s misperception of the virus’s exponential growth in linear terms and that overcoming this bias increases support for social distancing. Study 1 shows that American participants mistakenly perceive the virus’s exponential growth in linear terms (conservatives more so than liberals). Studies 2 and 3 show that instructing people to avoid the exponential growth bias significantly increases perceptions of the virus’s growth and thereby increases support for social distancing. Together, these results show the importance of statistical literacy to recruit support for fighting pandemics such as the coronavirus.

## Significance

Given the current lack of an effective vaccine to prevent coronavirus disease 2019 (COVID-19), one of the most effective ways to prevent the illness is social distancing. At the same time, a sizeable portion of the public fails to see the necessity of such measures. We identify one root cause for this: People mistakenly perceive the coronavirus to grow in a linear manner, underestimating its actual potential for exponential growth. We show that correcting this perceptual error significantly increases support for social distancing. This research shows the importance of statistical literacy among the general public for increasing support to fight the coronavirus using the most effective method currently available.

The threat that a pandemic such as coronavirus disease 2019 (COVID-19) poses is grave. Given the lack of a vaccine, the most effective available measure to fight and contain such viruses is social distancing. This can buy time for medical science to develop a treatment and can allow medical services the time to prepare for the ensuing surge in patients. Many countries across the globe have followed this strategy and have introduced social distancing measures. At the same time, sizeable opposition among politicians and the general population has delayed, prevented, or terminated early measures to increase social distancing. For example, toward the end of March 2020, a month in which, in the United States, the number of infections increased from a few dozen to 200,000 cases, one in four Americans opposed social distancing measures (1). Most strikingly, at the same time, even heads of state such as American President Trump or Brazilian President Bolsonaro repeatedly downplayed the growth of the virus and opposed social distancing measures (2, 3).

We propose that a root cause for why a sizeable portion of the people doubt the necessity of introducing such drastic measures is that people fail to recognize that the coronavirus can grow in an exponential manner, and, instead, erroneously perceive its growth in linear terms. A striking example of this is President Trump, who remained fixated on the low number of early infections in the United States and appeared not to realize how quickly this low number could spiral out of control (4). But, more in general, this prediction builds on literature showing that people, in general, have difficulty understanding exponential growth and erroneously interpret it in linear terms instead (5). This exponential growth bias is remarkably robust. It is shown when people extrapolate the growth of abstract numerical values, but it is also shown when growth is made easier to relate to—such as that of duckweed in a pond (6). The effect also occurs when correct estimates are incentivized, and it even shows up among those with greater mathematical sophistication or with relevant experience with growth processes (5, 7, 8). Making matters worse, people are overly confident in their ability to predict change. Particularly, those who have least knowledge about exponential growth and consistently apply linear thinking have particularly strong confidence in their erroneous forecasts (9, 10).

The current work tests the role of exponential growth bias in shaping the public’s view on social distancing to contain the coronavirus’s spreading. We first test, in study 1, whether people underestimate the exponential growth of the coronavirus. Moreover, we aim to show that the degree to which people show this bias depends, in part, on their political background. President Trump displayed exponential growth bias during the initial stages of the coronavirus outbreak, when he focused only on the initially low absolute numbers and ignored that exponential growth would quickly multiply those numbers (4). We test whether Republican supporters similarly show stronger exponential growth bias than liberals.

Building on this observation that some show an increased exponential growth bias in their perception of the coronavirus compared to others (due to incorrect information), we then test, in studies 2 and 3, whether the exponential growth bias can also be decreased with experimental instructions (that present correct information). Furthermore, we test whether such instructions can also increase support for social distancing. On the one hand, literature until now shows that the exponential growth bias is strongly resistant against instructions to correct for it (5–10). On the other hand, the coronavirus outbreak is a unique moment in history that directly impacts people’s deepest concerns about their lives and those of their loved ones. Exposure to news showing that the virus has grown remarkably quickly in other parts of the world may increase the availability of the concept of exponential growth or at least the perceptual readiness to understand it and its implications (11, 12). Indeed, earlier findings show that experience with exponential growth—such as in the case of hyperinflation in Israel in the early 1980s—can increase susceptibility to information that can help to overcome exponential growth bias (13). Based on this, we expected that people would be susceptible to information that can help them to correct for their biased perception of the coronavirus.

## Method and Results

To test these ideas, we conducted three studies in the second half of March 2020—a period in which the coronavirus in the United States increased particularly rapidly. This allows us to compare subjective growth perception and prediction with actual growth rates. Across these studies, we recruited American participants online via Amazon MTurk, a web-based tool for recruiting and paying participants to perform tasks. MTurk samples have been shown to be as representative of the US population as other sampling methods (14, 15). To avoid the most critical problem with MTurk samples—nonnaïveté (16)—participants were barred from taking part in more than one study. All three studies were conducted consistent with the Declaration of Helsinki, and all three are exempt from Institutional Review Board approval by guidelines of the German Psychological Society DGPS (Deutsche Gesellschaft für Psychologie) (17). Data and code are available at https://osf.io/xjwbg/ (18).

### Study 1.

**Fig. 1.**
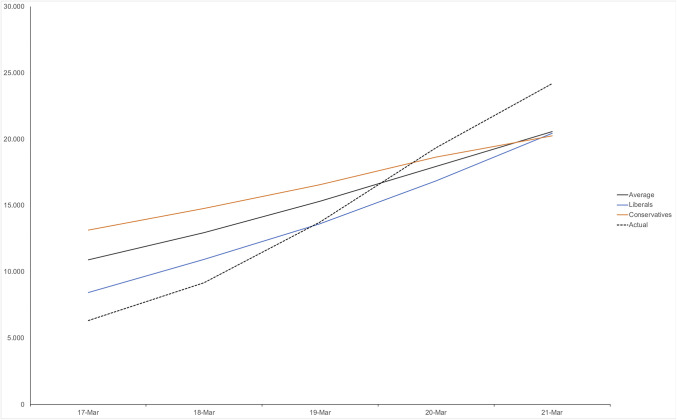
Study 1: Participants, on average, show exponential growth bias and underestimate the slope of the coronavirus growth curve over the past week, falsely believing the number to be higher early in the week than it was (gray, dashed line shows actual total number). Conservatives (red) do so more strongly than liberals (blue) (continuous data split across the neutral midpoint, for presentation purposes).

After providing consent, participants guessed the total number of coronavirus cases over the past 5 d, from Tuesday, March 17 to Saturday, March 21. As expected, participants displayed exponential growth bias. Although some participants accurately included exponential growth in their estimates, thus producing an overall significant quadratic trend (*F* = 18.78, *P* < 0.0001), its size was dwarfed by the strong linear trend (*F* = 470.55, *P* < 0.0001), meaning that participants’ averaged estimates of the virus’s growth could, for practical purposes, be described as linear (Fig. 1, dark gray line). Comparing participants’ estimates against linear and quadratic trends in the actual data of the virus’s growth (Fig. 1, dashed black line), drawn from the Worldometer COVID-19 database (19), we found that participants underestimated both the virus’s linear (*P* < 0.0001) and exponential growth (*P* < 0.0001). Note that, as a result of their failure to see exponential growth, participants did not simply underestimate the number of cases throughout the observed time period. In fact, they overestimated the number of known coronavirus cases in the first 3 d of the week (all *P* < 0.0001) and underestimated the number in the last 2 d of the week (both *P* < 0.0001). On average, they underestimated the actual growth of the virus’s over that time period by 45.7% (*P* < 0.0001).

As also expected, this tendency to underestimate exponential growth was not fixed but instead depended on participants’ political ideology (*P* < 0.0001), which we measured using a validated continuous scale (20). A significant ideology × linear trend (*P* < 0.0001) suggested that conservatives were more likely to underestimate the virus’s absolute growth compared to liberals. A significant ideology × quadratic trend (*P* = 0.006) showed that conservatives also underestimated the exponential nature of that growth more than did liberals (Fig. 1; data split across the neutral midpoint between liberals and conservatives, for presentation purposes). Again, note that, compared to liberals, conservatives did not underestimate but overestimated the number of virus infections in the first 3 d of the week (all *P* < 0.001). In other words, compared to liberals, conservatives did not underestimate the problem (defined as number of infections) per se, but underestimated its exponential growth.

### Study 2.

Our next aim was to test whether this incorrect perception of the coronavirus’s growth could be corrected by instructing participants about exponential growth and whether doing so also affects support for social distancing measures. To do so, we repeated the design of study 1, but randomly assigned participants to one of two conditions. After providing consent, participants in the experimental condition received the following instructions, that were based on the virus’s recent developments (19):

Please keep in mind that many people forget that the speed by which the corona virus spreads, increases each day. In other words, when making these guesses, many people erroneously think that the coronavirus cases have increased at a steady and constant pace. In reality, in the USA (as in almost all other countries) the number of corona patients doubles and keeps doubling every three days.

**Fig. 2.**
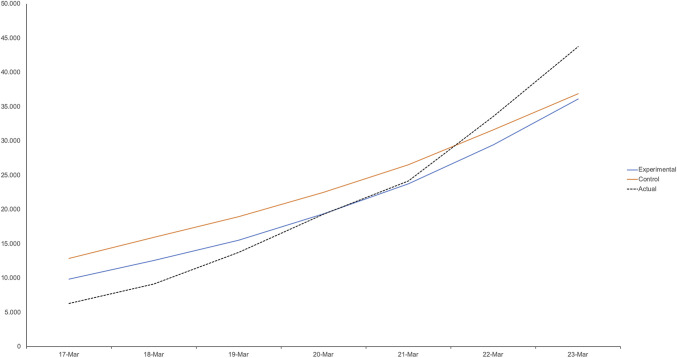
Study 2: Instructing participants to correct for exponential growth bias (blue) partially reduces the biased perception the coronavirus growth, compared to a control condition (red). Dashed line shows actual total number.

In the control condition, participants did not receive these instructions. Next, participants guessed the number of coronavirus cases between Tuesday, March 17 and Monday, March 23. These experimental instructions affected participants’ perceptions of the growth of the virus (*P* = 0.003). Following this up by testing interactions between condition and polynomial contrasts, we found no significant condition × linear trend interaction (*P* = 0.104), but only a significant condition × quadratic trend interaction (*P* = 0.001), suggesting that the experimental instruction primarily corrected participants’ misunderstanding of the virus’s exponential growth (Fig. 2). Consistent with predictions, participants in the experimental condition were also significantly more supportive of social distancing than participants in the control condition (*P* = 0.019).

### Study 3.

**Fig. 3.**
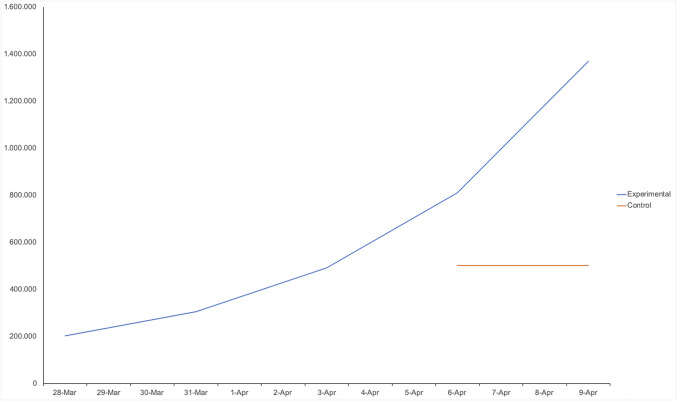
Study 3: Instructing participants to estimate the number of cases at the end of 2 wk by making four intermediate steps helps them understand the potential implications of exponential growth (blue), compared to a control condition (red).

An even more effective way to increase support for social distancing (compared to changing people’s beliefs about past growth) may be correcting beliefs about the virus’s future growth. After providing consent, all participants read the current estimated number of coronavirus infections in the United States and the current statistic that it doubles every 3 d (19). Next, all participants guessed the development of the virus’s spread over the next 15 d. In the experimental condition, participants were instructed to arrive at their estimate in five steps, first guessing the number of active coronavirus cases in four intermediate steps, each 3 d apart. Because this time frame matched the statistic (provided to all participants) that the number of cases doubles every third day, this helped participants understand the implications of exponential growth. In the control condition, participants instead made an immediate estimate of the number of cases after 2 wk. Importantly, these participants received the same statistical information (including the current number of cases and its speed of doubling) but were not instructed to make the four intermediate guesses. As expected, participants in the experimental condition produced 173% higher final estimates of the number of known cases of coronavirus infection after 2 wk, than control participants (*P* < 0.0001; Fig. 3). Furthermore, being helped to realize the potential implications of exponential growth in the near future, participants in the experimental condition were significantly more supportive of measures to increase social distancing and a lockdown than control participants (*P* = 0.024). Finally, a mediation analysis showed that the latter effect of condition on support for social distancing was statistically mediated by the former effect on participants’ final estimates (*P* = 0.011).

## Discussion

Across three studies, we found evidence of exponential growth bias in people’s perceptions of the coronavirus’s spread, meaning that people erroneously perceive the virus’s exponential growth in largely linear terms. This effect was stronger among conservatives than liberals (study 1), who followed President Trump’s incorrect remarks about the virus. This shows the danger of politicians’ downplaying of the virus. Furthermore, we found that participants can be helped to correct for the exponential growth bias in estimating the virus’s development in the recent past (study 2) and immediate future (study 3). These interventions not only help overcome exponential growth bias, but they also significantly increase support for social distancing—the most effective available way to prevent spreading of the coronavirus.

Our results stand in contrast to earlier literature that shows that the exponential growth bias is difficult to overcome (5–10, 21). Instead, in our studies, a three-sentence instruction not to make the mistake (study 2) or an instruction to estimate through four intermediate steps (study 3) effectively reduced the bias. A difference between our and earlier studies is that we focused on a threat with great personal relevance and media presence, which likely increases subjective availability and thus estimated probability of the risk. This possibly increases the readiness to understand exponential growth when instructed about it and reduces the underestimation of exponential growth (11–13).

These findings demonstrate the real-life implications of exponential growth bias. Earlier work shows the bias affects households’ financial decisions (22), but the current findings show that it also influences political opinions about matters of life and death. Given that social distancing is the most effective way to combat the coronavirus currently available, these findings are of great impact. More generally, our findings show the importance of statistical literacy and echo calls to improve that skill among the general public (23, 24).
